# Sand flies (Diptera, Psychodidae, Phlebotominae), vectors of *Leishmania* protozoa, at an Atlantic Forest Conservation Unit in the municipality of Nísia Floresta, Rio Grande do Norte state, Brazil

**DOI:** 10.1186/s13071-016-1352-5

**Published:** 2016-02-11

**Authors:** Marcos Paulo Gomes Pinheiro, Marcel Miranda de Medeiros Silva, João Batista Silva Júnior, José Hilário Tavares da Silva, Maria de Lima Alves, Maria de Fátima Freire de Melo Ximenes

**Affiliations:** Laboratório de Entomologia, Centro de Biociências, Universidade Federal do Rio Grande do Norte, Avenida Senador Salgado Filho, 3000 Natal, Rio Grande do Norte Brazil

**Keywords:** Sand flies, *Lutzomyia*, *Psychodopygus*, *Nyssomyia*, Visceral leishmaniasis, American tegumentary leishmaniasis, Atlantic forest

## Abstract

**Background:**

Sand flies are insect vectors of protozoa from the genus *Leishmania*, causative parasites of visceral and American tegumentary leishmaniases. The present study discusses the bioecological aspects of sand fly species, transmitters of *Leishmania* protozoa, in different ecotopes of an Atlantic Forest Conservation Unit located in the metropolitan region of Natal, Rio Grande do Norte state, Brazil.

**Methods:**

Two monthly captures were made in 1 year, using CDC light traps, in two anthropized and two preserved environments.

**Results:**

A total of 2936 sand flies belonging to the following ten species were captured: *Evandromyia walkeri*, *Evandromyia evandroi*, *Psychodopygus wellcomei*, *Sciopemyia sordellii*, *Psathyromyia brasiliensis*, *Lutzomyia longipalpis*, *Evandromyia lenti*, *Psathyromyia shannoni*, *Nyssomyia whitmani* and *Nyssomyia intermedia*. The most common species was *E. walkeri* (77.6 %), followed by *E. evandroi* (17.5 %). Forest was the site with the greatest abundance (32.4 %), followed by bamboo grove (26.3 %).

**Conclusions:**

Sand flies were generally more abundant in the rainy season and *L. longipalpis*, a vector species of *Leishmania infantum,* was adapted to anthropized environments. It was confirmed that *P. wellcomei*, a vector of *Leishmania braziliensis* in Amazônia, is a species associated with more preserved environments, and occurs only in the rainy season.

## Background

Sand flies are insects responsible for transmitting the causative parasites of American tegumentary (ATL) and visceral leishmaniases (VL), a serious health problem and among the major human diseases transmitted by insect vectors that can cause mutilation, incapacity and death [1, 2].

Although cutaneous and mucosal leishmaniasis are endemic to 18 countries in the Americas, with cases ranging from Mexico to Argentina, most cases are concentrated in Brazil and the Andean subregion [3].

VL is endemic to Latin America and most cases occur in Brazil, with risk factors also found in Argentina, Bolivia, Colombia, Costa Rica, El Salvador, Guatemala, Honduras, Mexico, Nicaragua and Venezuela [3].

In Brazil, the etiologic agent of VL is the protozoan *Leishmania infantum* and the vector is the sand fly *Lutzomyia longipalpis* [4]. ATL exhibits different causative agents and vectors, depending on the region of the country [5–12]. Among the sand flies considered vectors of ATL in Brazil, *Nyssomyia whitmani, Nyssomyia intermedia*, *Nyssomyia umbratilis*, *Migonemyia migonei, Psychodopygus complexa* and *Psychodopygus wellcomei* occur in the Northeast [6, 12–15]. Effective measures to control the vectors are increasingly necessary.

Leishmaniasis transmission occurs in all regions of Brazil, with the largest number of VL cases recorded in the Northeast (53.6 % of confirmed cases), where the disease, formerly restricted to rural areas, has expanded to periurban and urban regions [4, 16]. In recent years, it has spread to urban areas throughout the country, and is continuously advancing in 21 states [17].

ATL occurs in all regions of Brazil, with outbreaks in the South, Southeast, Midwest, Northeast and North, primarily in the Amazon, where it is associated with colonization processes in areas deforested to construct roads, new urban centers and expand agricultural activities [18, 19].

In Rio Grande do Norte, cutaneous leishmaniasis is caused by *Leishmania braziliensis* [5] and occurs predominantly in the highland area of the state, primarily in municipalities at altitudes between 500 and 700 m. VL is more common in the Metropolitan region, where 37 % of the cases were recorded between 2007 and 2014, but can also be found in municipalities in the Western region of the state, which has reported a significant number of cases in recent years [5, 20–22].

This study aimed to determine the composition of sand fly fauna in different ecotopes of an Atlantic Forest fragment, in order to broaden knowledge of the vector species, thereby contributing to epidemiological surveillance and control in the state.

## Methods

### Study area

The study was conducted in the National Forest (FLONA) of Nísia Floresta, an Atlantic Forest Conservation Unit located in the municipality of Nísia Floresta, Rio Grande do Norte state, Brazil. It is situated 30 km from the state capital, encompasses an area of 307,839 km^2^, with a population of 26,208 inhabitants in 2014, according to the Brazilian Institute of Geography and Statistics [23].

The Nísia Floresta FLONA is located in a region historically exploited for sugarcane monoculture. It is a conservation and research unit with 168.84 ha of different exotic and native Atlantic Forest plant cover [24].

**Fig. 1:**
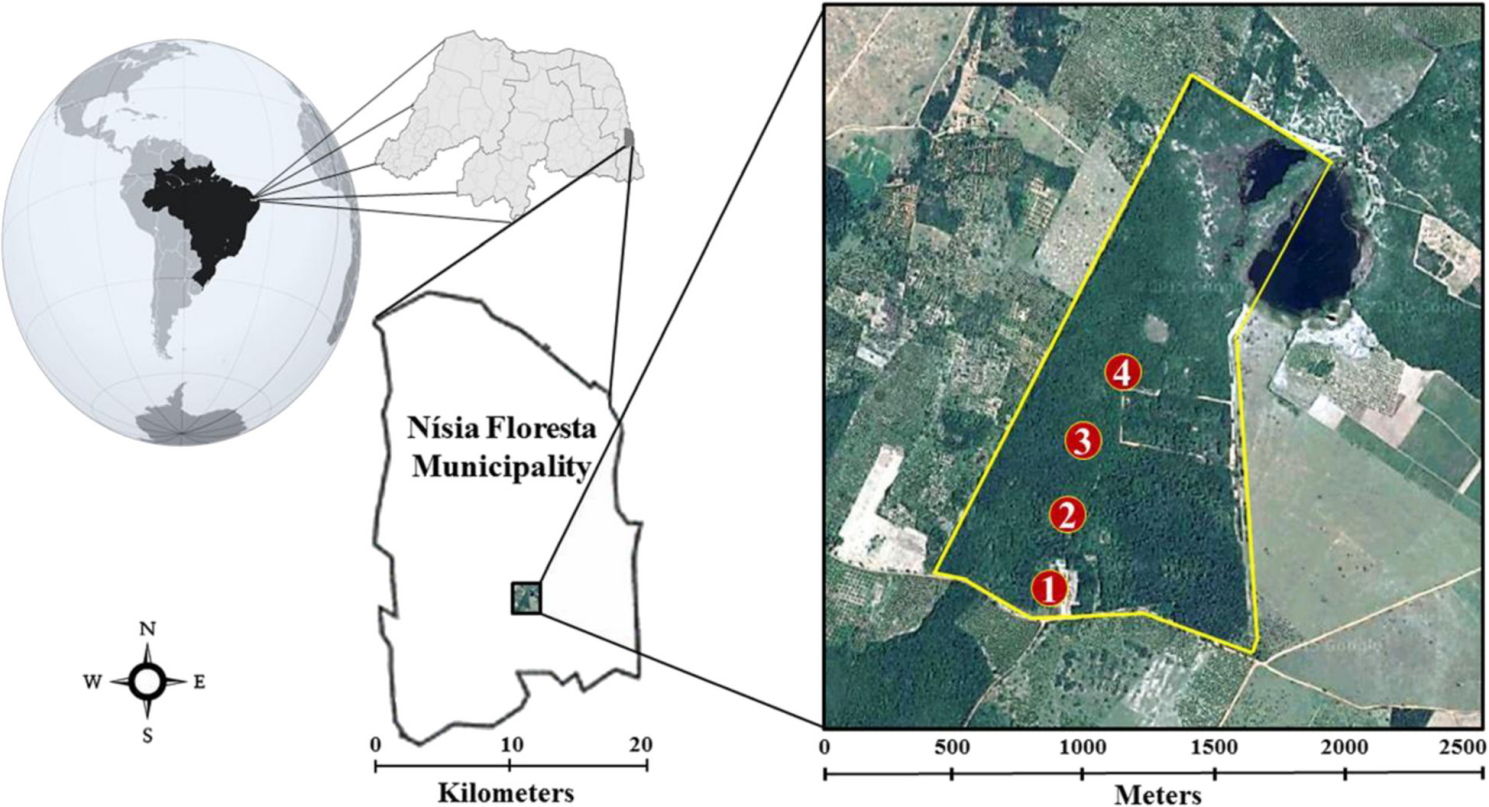
Location of the municipality of Nísia Floresta and the Conservation Unit where collections took place

Collections occurred at four specific points, denominated bamboo (1), trail (2), forest (3) and pinery (4), approximately 300 m apart (Fig. 1).

Bamboo (S06°05’13.9”/W035°11’09.1”) and trail (S06°05’09.2”/W035°11’06.5”), the closest points to the FLONA administration building, are more affected by anthropic activity.

The former (1) is significantly impacted and characterized by a number of bamboo plants (Poaceae: Bambusoideae) in a 900 m^2^ clearing containing litter and decomposing branches, in addition to discarded plant trimmings.

The latter (2) contains native trees such as *Tabebuia* sp. (ipê), *Caesalphinia echinata* (Brazilwood), *Caesalpinia ferrea* (Brazil ironwood), *Bauhinia forficata* (Brazilian orchard tree), among others, as well as exotic varieties such as *Eucaliptus* sp. (eucalyptus) and *Pinus* sp. (pine tree) [24] on a trail about 50 m from a soccer field located between the administration building and the forest.

The Atlantic Forest fragment (S06°04’57.7”/W035°10’58.7”) and the pinery (S06°04’50.2”/W035°10’57.8”) are the best preserved areas of the forest, with little anthropic activity.

The third area (3), denominated forest, consists of medium-sized and large native trees, between 10 and 25 m high, whose main species are *Bowdichia vigilioides* (sucupira), *Lecythis pisonis* (cream nut), *Buchenavia* sp. (mirindimba), *Tapirira guianenis* (cupiúba), *Myrcia* sp. (pau mulato), *Coccoloba* sp. (cauaçu), *Tocoyena* sp. (juruparana), *Hymenaea* sp. (jatobá), *Cassia apoucovita*, *C. ferrea* (pau ferro), *Tabebuia* sp. (ipê), *C. echinata* (Brazilwood), *B. forficata* (Brazilian orchard tree), among others. It is considered a fragment of the Semideciduous Seasonal Forest at an advanced stage of regeneration, covering around 80 ha, 45.22 % of FLONA’s total area [24].

The fourth area (4), the pinery, is located in the experimental area for Atlantic Forest regeneration, involving the planting of exotic pine trees (*Pinus* sp.) [24].

### Sandfly captures and identification

Collections were carried out twice a month from May 2012 to April 2013, using a CDC (Center for Disease Control) light trap placed 1 m above the ground, activated at 5 pm and removed at 7 am the following morning.

The insects were sacrificed and screened in the Entomology Laboratory of Universidade Federal do Rio Grande do Norte, at a temperature of −20 ° C.

The sand flies were then cleared in 10 % potassium hydroxide (KOH), mounted and observed under optical microscope for morphological identification, based on phylogenetic classification proposed by Galati [25, 26]. They were then cataloged and stored in the Professor Adalberto Antônio Varela-Freire Entomological Collection of Universidade Federal do Rio Grande do Norte.

### Statistical data analysis

Analysis to determine and compare diversity between environments was conducted using the Shannon-Wiener Diversity Index (H’) [27], calculated with Ecological Methodology 5.2 software [28].

The index of species abundance (ISA) and standardized index of species abundance (SISA) [29] were applied to analyze abundance data. The ISA values were determined and converted into SISA values between 0 and 1, using Microsoft Office Excel 2007.

The results were compared with rainfall, relative humidity and temperature data obtained from the National Meteorology Institute [30].

## Results

A total of 2936 sand flies were collected from six genera belonging to the following ten species: *Evandromyia walkeri*, *Evandromyia evandroi*, *P. wellcomei*, *Sciopemyia sordellii*, *Psathyromyia brasiliensis*, *L. longipalpis*, *Evandromyia lenti*, *Psathyromyia shannoni*, *N. whitmani* and *Nyssomyia intermedia* (Table 1).

**Table 1:** Sand fly species collected in each ecotope, results of SISA and H’

Species	Ecotopes				Total	%	SISA	Ranking
	Pinery	Forest	Trail	Bamboo				
*Evandromyia walkeri*	456	779	473	589	2297	77.60 %	1.00	1
*Evandromyia evandroi*	138	118	80	159	495	17.50 %	0.87	2
*Psychodopygus wellcomei*	11	32	8	3	54	1.90 %	0.82	3
*Sciopemyia sordellii*	4	13	6	3	26	0.86 %	0.81	4
*Lutzomyia longipalpis*	1	3	6	9	19	0.64 %	0.80	5
*Evandromyia lenti*	2	3	4	1	10	0.34 %	0.79	6
*Psathyromyia brasiliensis*	10	6	6	0	22	0.73 %	0.56	7
*Psathyromyia shannoni*	0	1	1	7	9	0.30 %	0.54	8
*Nyssomyia whitmani*	0	3	0	0	3	0.10 %	0.04	9
*Nyssomyia intermedia*	0	1	0	0	1	0.03 %	0.04	10
Total	622	959	584	771	2936	100 %	-	-
%	21.5 %	32.4 %	19.8 %	26.3 %	100 %	-	-	-
H’	1.097	1.008	0.993	0.978	-	-	-	-

The environment containing the largest number of sand flies collected was the forest, accounting for 32.4 % of the specimens, followed by bamboo and pinery, with 26.3 % and 21.5 % respectively, and trail with 19.8 % (Table 1).

**Fig. 2:**
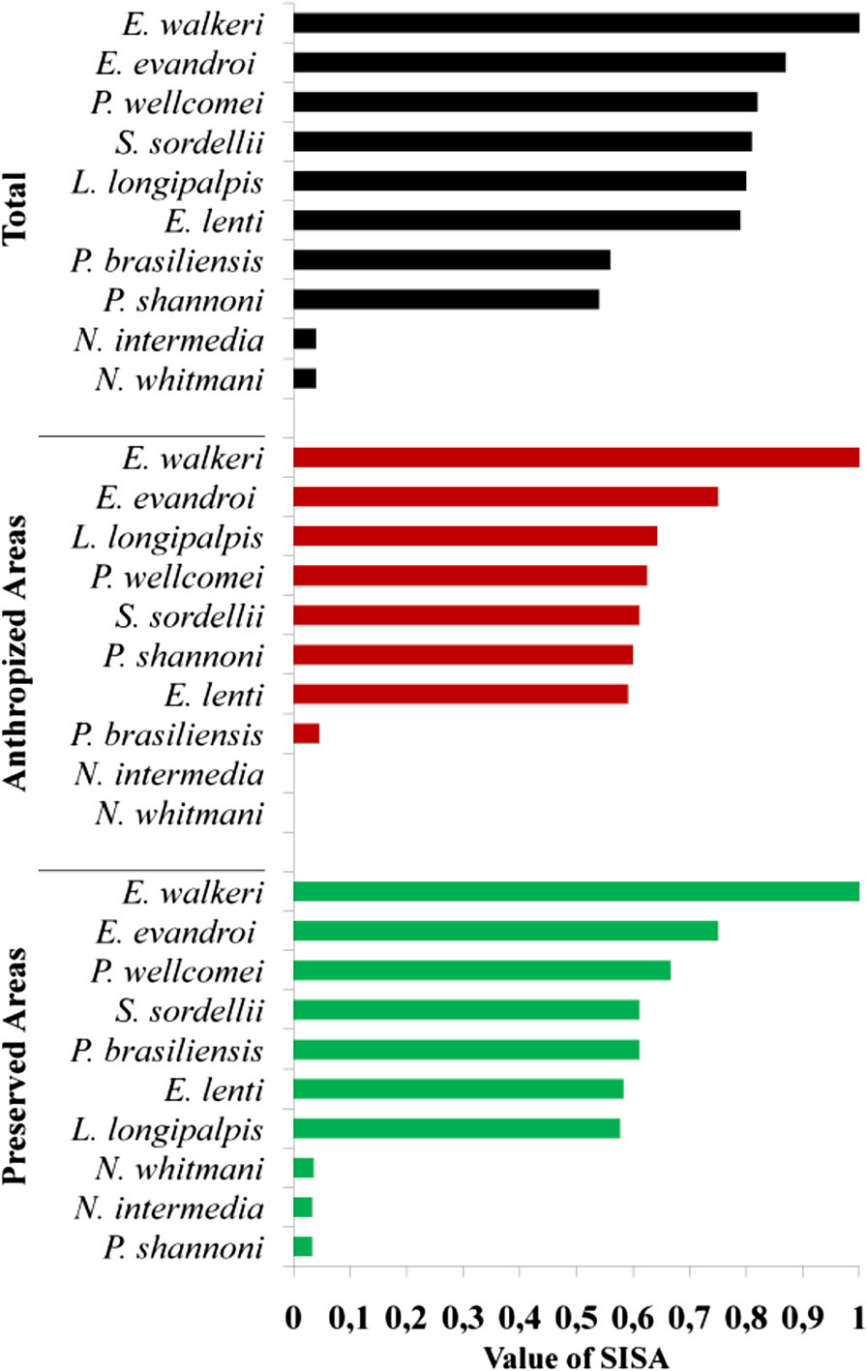
Standardized index of total species abundance (SISA), in anthropized (bamboo and trail) and forest environments (forest and pinery)

*E. walkeri* was the most abundant in each ecotope, corresponding to 77.6 % of all sand flies collected (SISA = 1.0), followed by *E. evandroi* with 17.5 % (SISA = 0.87) (Fig. 2). The least abundant species were *N. whitmani* (SISA = 0.04) and *N. intermedia* (SISA = 0.04) (Table 1).

The collection site with the greatest diversity, according to the Shannon Diversity Index (H’), was the pinery (H’ = 1.097), followed by forest (H’ = 1.008) (Table 1).

**Fig. 3:**
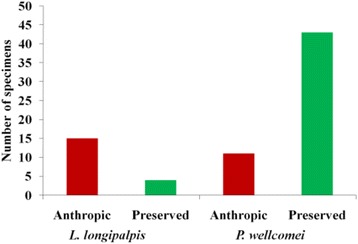
Occurrence of *L. longipalpis* and *P. wellcomei* in anthropized (bamboo and trail) and forest environments (forest and pinery)

*P. wellcomei* was the most abundant at points of less anthropic activity, while *L. longipalpis* was the most common in areas of significant human intervention (Table 1) (Fig. 3).

The largest number of sand flies were collected in May 2012 (33.7 % of the captures), and the lowest in April 2013, with only five specimens, four *E. evandroi* and one *E. walkeri* (Table 2).

**Table 2:** Sand fly species collected monthly at the Conservation Unit

Species	Month												Total
	May	Jun	Jul	Aug	Sep	Oct	Nov	Dec	Jan	Feb	Mar	Apr	
*Evandromyia walkeri*	863	156	299	86	80	90	142	68	92	300	120	1	2297
*Evandromyia evandroi*	87	29	63	19	36	32	34	35	40	80	36	4	495
*Psychodopygus wellcomei*	25	5	9	9	2	0	0	0	0	3	1	0	54
*Sciopemyia sordellii*	10	0	4	2	3	4	1	0	0	1	1	0	26
*Psathyromyia brasiliensis*	8	0	5	5	4	0	0	0	0	0	0	0	22
*Lutzomyia longipalpis*	7	2	1	0	6	0	1	0	0	2	0	0	19
*Evandromyia lenti*	5	0	0	1	0	1	0	2	1	0	0	0	10
*Psathyromyia shannoni*	1	0	1	7	0	0	0	0	0	0	0	0	9
*Nyssomyia whitmani*	0	0	2	0	1	0	0	0	0	0	0	0	3
*Nyssomyia intermedia*	1	0	0	0	0	0	0	0	0	0	0	0	1
Total	1007	192	384	129	132	127	178	105	133	386	158	5	2936

**Fig. 4:**
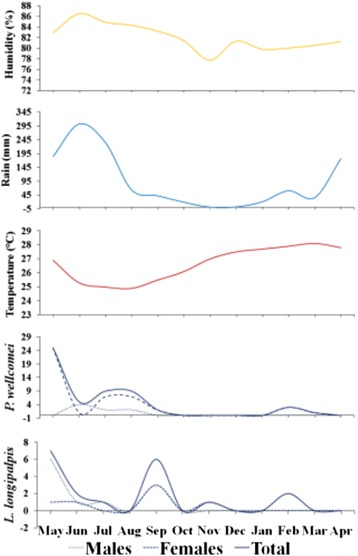
Annual distribution of *P. wellcomei* and *L. longipalpis* at the Conservation Unit and climatic variables (relative humidity, rainfall and mean temperature)

In all collection months, the species with the highest density were *E. walkeri* and *E. evandroi* (Table 2). *P. wellcomei* was the third most collected species in May, June, July, August and February (Table 2), months when considerable rainfall occurs (Fig. 4). There was a tendency for a larger number of sand flies of most species in the rainy months (Table 2).

## Discussion

The most abundant species collected were *E. walkeri* and *E. evandroi*, as yet not identified as vectors of *Leishmania*. However, the presence of vectors such as *L. longipalpis*, *N. whitmani*, *N. intermedia* and *P. wellcomei* is noteworthy.

Among sand fly vectors, there is an association between *L. longipalpis*, transmitter of *L. infantum* and modified environments, since most specimens were collected in this area. This is corroborated by numerous studies reporting the occurrence of these species in modified environments, evidencing their anthropophilic behavior [4, 31, 32].

*N. whitmani* and *N. intermedia*, species confirmed as vectors of *L. braziliensis* in forest environments in the Northeast [7, 13], and *P. wellcomei*, a vector species in Amazônia [33–35], have demonstrated a preference for more preserved areas of the Conservation Unit. *P. wellcomei* and *N. whitmani* are predominantly forest dwellers in the study areas, given that most of the captures of both species occurred in such environments. However, it is important to underscore the easy adaptation of *N. whitmani* to anthropized environments [36].

The study area exhibited a difference between the habitats of vector species of causative agents of ATL and *L. longipalpis*, transmitter of *L. infantum*, likely due to phytogeographic and biological factors. This difference was also suggested in studies conducted by other authors in Bahia state, where *L. longipalpis* occurs in areas of the Caatinga or in those with anthropic activity, whereas *N. whitmani* and *N. intermedia* are found in tropical forests [32, 37, 38]. This shows that some regions exhibit a likely association between ATL-transmitting species and forest environments, whereas *L. longipalpis*, a VL transmitter, is associated with Caatinga environments or degraded areas with anthropic activity.

The fact that *L. longipalpis* is more common in anthropized environments confirms the anthropophilic behavior of the species and is undoubtedly related to cases of human VL in the metropolitan region of Natal. A total of 799 cases of VL were diagnosed in Rio Grande do Norte state between 2007 and 2014, 297 in the metropolitan region [21]. Thus, the greater frquency of *L. longipalpis* in anthropized environments confirms the invasive potential of this species, as well as its success as a vector in urban areas.

*P. wellcomei*, the third most abundant species in this study, was initially found in the Brazilian Amazon [39] and later in the Northeast, in forests in Ceará state [33], forest and peridomiciliary environments in Maranhão state [13, 40], and in forest environments of metropolitan regions in the states of Pernambuco [35] and Rio Grande do Norte [15].

Although studies on *P. wellcomei* in Northeastern Brazil are not sufficient to consider it as a vector in the region [33–35], the occurrence of this species is extremely relevant, since it is a confirmed vector of *L. braziliensis* in Amazônia [39, 41] and exerts intense anthropofilia even in the daytime [42]. The endemic area of cutaneous leishmaniasis in Rio Grande do Norte is in the highland region of the state, the occurrence of this species in the metropolitan region, with greater abundance during some months of the year, reinforces the need for surveillance, given that residents, workers and researchers are in or near the forest on a daily basis.

In this study, *P. wellcomei* was associated with rainy months, corroborating the pattern described for the Amazon [8]. This leads us to suspect the possibility of diapause during the dry season, as reported in studies carried out in the Amazon [43]. Diapause seems to be a strategy adopted by sand flies to survive during adverse or extreme conditions [44].

Even though the study area is not endemic for ATL, it is important to register the occurrence of a small number of *N. whitmani*. This anthropophilic species is a vector of *L. braziliensis* in a number of areas in Brazil [9, 10, 45], including the Northeast [7], and its importance as a vector depends on its adaptation to the features of local ecotopes [46].

It is important to underscore the urban expansion at the edge of the Conservation Unit, where there has been an increase in residential condominiums. This interference may lead to the adaptation of vectors to new environments and alternative food sources, and at certain moments favor transmission cycles of the protozoan, as observed after colonization or deforestation [47–50].

The presence of predominantly forest-dwelling sand flies reported in this study indicates a certain persistence in survival, and consequent transmission potential, despite the growing environmental degradation that has occurred surrounding the study area. It is important to highlight the increasing adaptation of vector species to environments modified by man [51], which may contribute to maintaining the vector potential of these species.

Even though *E. walkeri* and *E. evandroi* are dominant in areas of the Conservation Unit with significant anthropic activity (bamboo and trail), the concomitant presence of *P. wellcomei*, a species more adapted to forest environments, and *L. longipalpis*, an urbanized species, is relevant. In this respect, the possibility of sand flies’ seeking food in inhabited areas is important, as suggested in another study carried out in the metropolitan region of Natal, where these vectors may have adapted to feeding on exotic plants, whose monosaccharides coincided with those detected in plants such as *Eucalyptus* sp. (eucalyptus) and *Pennisetum pupureum* (elephant grass) [52].

*P. shannoni* was captured at three points: bamboo, trail and forest. In the United States, this species is considered a vector of *Vesiculovirus*, the cause of vesicular stomatitis [53], and a number of studies indicate that *L. infantum* develops well in this sand fly [54]. It is also hypothesized that the presence of infected dogs in occurrence areas of *P. shannoni* could give rise to epidemic cycles [55].

Given that expansion and urbanization of leishmaniases results from interactions between hosts and the protozoan parasite, as well as environmental degradation, studies aimed at analyzing the bioecology of vector species and their potential to adapt to new environments are sources of information that may contribute to the epidemiological surveillance of leishmaniases.

## Conclusions

The rainiest period coincided with the highest occurrence of sand flies, and the greatest abundance and diversity was recorded in a preserved forest area.

There was a difference in species composition between the most anthropized and preserved areas.

It was confirmed that *P. wellcomei* is a species adapted to the rainiest period of the year and to forest environments, especially preserved areas. However, this species has been able to adapt to degraded environments, since in this study it also appears in environments with greater anthropic activity.

*L. longipalpis* seemed more adapted to areas with anthropic intervention; however, it can also be found in preserved environments.

## Abbreviations

ATL: American tegumentary leishmaniasis
CDC: center for diseases control
FLONA: national forest
VL: visceral leishmaniasis
